# Effects of water flow conditions on soil properties and microbial communities in the lower Heihe river Basin, Northwest China

**DOI:** 10.1186/s12866-025-04393-7

**Published:** 2025-10-07

**Authors:** Shumin Wang, Yongyong Zhang, Wenrong Kang, Shue Wei, Jianhua Xiao

**Affiliations:** 1 National Field Science Research Station of Farmland Ecosystem in Linze, State Key Laboratory of Ecological Safety and Sustainable Development in Arid Lands, Northwest Institute of Eco-Environment and Resources, Chinese Academy of Sciences, Lanzhou, 730000 Gansu China; 2 University of Chinese Academy of Sciences, Beijing, 100049 China

**Keywords:** Flow condition, Soil microbial community, Environment factor, Inland river

## Abstract

**Background:**

Effective hydrological management in arid ecosystems requires a comprehensive understanding of how water flow conditions influence biota and ecosystem processes. However, the responses of soil microbial communities remain poorly understood. This study aims to evaluate whether, and to what extent, a 20-year difference in flow regimes affects soil microbial diversity, composition, and function. The composition of the soil microbial community and the factors shaping it were investigated in two arid riparian zones (East River and West River) of the lower Heihe River Basin using 16 S rRNA amplicon high-throughput sequencing.

**Results:**

The dominant phyla of the bacterial, fungal, and archaeal communities were Proteobacteria, Ascomycota, and Euryarchaeota, respectively. Co-occurrence network analysis revealed greater network connectivity and stability in the West River, suggesting enhanced mutualistic interactions and physiological acclimation strategies in response to low-flow conditions. Microbial-soil correlations varied with flow condition: bacterial and fungal communities under high-flow conditions were associated with bulk density and available nitrogen, whereas low-flow communities were more influenced by available potassium. High-flow conditions also strengthened the correlation between microbial communities and nitrogen-related functions. Functional prediction showed that chemoheterotrophy was the dominant bacterial function, and bacterial and archaeal communities were partially involved in the nitrogen cycle.

**Conclusions:**

Changes in flow regimes slightly modulated microbial community composition and diversity. However, microbial functions appeared to respond more strongly to hydrological factors than microbial diversity. Thus, alterations in microbial structure and function were jointly influenced by hydrological conditions and the availability of nutrients in arid riparian zones.

**Supplementary Information:**

The online version contains supplementary material available at 10.1186/s12866-025-04393-7.

## Introduction

Rivers play a critical role in sustaining water-limited arid and semi-arid ecosystems by maintaining key hydrological, geological, and ecological processes [1]. The complex interplay between environmental components—such as the combined effects of precipitation and snowmelt events—leads to temporal variability in river discharge patterns [2]. In addition to these natural annual fluctuations, human activities have increasingly altered river hydrology. Artificial water diversion has enabled the reallocation of water resources for ecological, societal, and economic purposes, as seen in the Murray-Darling Basin (Australia), Colorado River (USA), and Heihe River (China) [3–5]. Because a river’s flow regime governs both in-stream and floodplain ecosystem dynamics, flow alterations are rarely without consequence [1].

Hydrological regimes are closely linked to changes in soil properties, including the structure and composition of soil microbial communities [6, 7]. Most existing studies on this topic have primarily focused on wetland ecosystems [7, 8]. In contrast, relatively little attention has been paid to the soil microbes adjacent to rivers in arid environments. However, a few studies have indicated that changes in flow regimes can significantly affect microbial abundance and community structure [9, 10]. For instance, Zeglin et al. [11] reported that microbial enzyme activity was higher in moist near-stream margins than in dry soils. Similarly, research in a tributary of the Yellow River found that bacterial abundance varied significantly across hydrological periods, with increases observed during low-flow seasons [12]. Moreover, the edaphic properties typical of arid soils may exert continued biochemical control over microbial function [11]. Evidence from wetland ecosystems also suggests that Gram-positive bacteria often dominate microbial communities, regardless of differences in hydrological regimes [13]. Nonetheless, identifying dominant ecological processes in complex environments such as soil remains a major challenge [10, 13].

Despite growing recognition of the ecological importance of hydrological regimes, few studies have investigated how soil microbial communities respond to human-induced hydrological changes. The lower reaches of the Heihe River in arid Northwest China experienced significant water shortages and environmental degradation during the 1980 s [14]. In response, the Ecological Water Diversion Project (EWDP) was launched in 2000 to relocate water resources and restore degraded ecosystems, resulting in substantial changes to the region’s hydrological regimes. Subsequent studies documented noticeable shifts in riparian vegetation, land use, and groundwater levels [15, 16]. Vegetation index data revealed a significant greening trend in the downstream, near-river regions [5, 17]. While many studies have explored how these hydrological changes influence plant communities, few have examined their effects on soil microbial communities in the post-EWDP era.

**Fig. 1:**
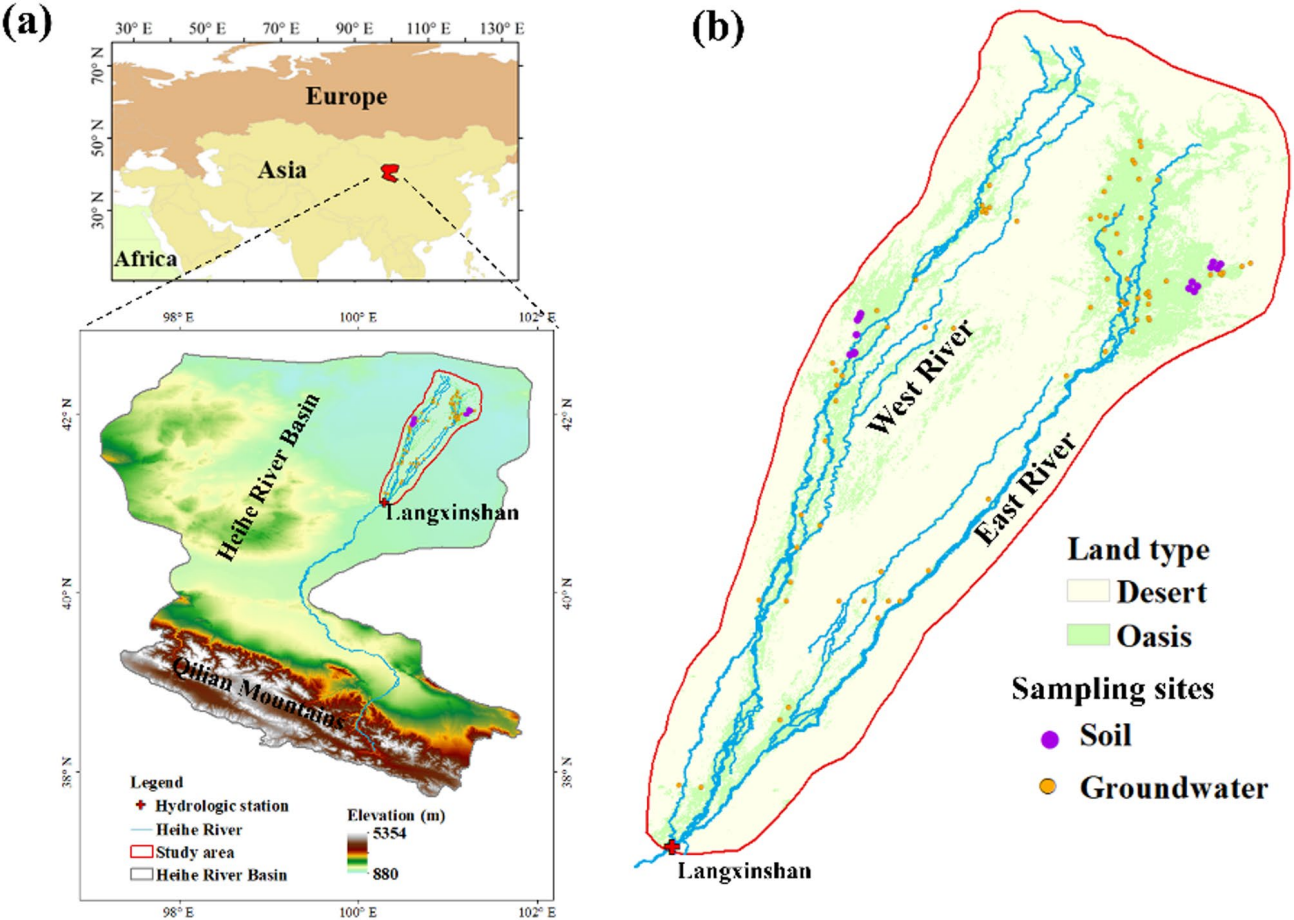
The location of study area (a) and sampling sites (b)

At Langxinshan hydrological station, the Heihe River divides into two branches: the East River and the West River (Fig. 1). These branches exhibit distinct low-flow and high-flow regimes. The present study aimed to evaluate whether, and to what extent, the 20-year difference in flow regimes has affected soil physicochemical properties and microbial community structure in the lower reaches of the Heihe River. We hypothesized that: (i) both soil characteristics and microbial communities vary with flow regime; (ii) hydrological background influences microbial stability differently; and (iii) specific soil nutrients mediate microbial function under varying flow conditions.

## Materials and methods

### Study area

The study was conducted in the lower reaches of the Heihe River, located in the arid zone of northwestern China (Fig. 1a). This region experiences a continental climate characterized by extremely low annual precipitation (approximately 35 mm) and high annual potential evaporation (up to 2300 mm) [5]. The Heihe River originates from rainfall and snowmelt in the Qilian Mountains (Fig. 1a) and branches into two distributaries—the East River and the West River—at the Langxinshan hydrological station (Fig. 1b). The main hydrological contrast between the two branches lies in their distinct low-flow and high-flow regimes. Due to the region’s aridity and limited rainfall, the Heihe River serves as the primary water source supporting environmental equilibrium [14]. Vegetation in the lower reaches aligns along the river course, transitioning from forests near the runoff to shrubs in more distal zones as water availability decreases. The dominant vegetation types are *Populus euphratica* and *Tamarix ramosissima*.

### Hydrological data

Hydrological and surface water datasets were used in this study. Streamflow and discharge data were obtained from the Heihe River Water Resources Bulletin, consisting of annual records from 2000 to 2019 at the Langxinshan hydrological station, where streamflow from both East and West Rivers was recorded. Groundwater data comprised 79 field observations in 2019, including 51 from the East River region. Surface water dynamics were derived from the JRC Global Surface Water dataset (JRC/GSW1_4/YearlyHistory), developed by the European Commission Joint Research Centre (EC JRC) and accessed via Google Earth Engine [18]. A 4-km buffer zone around the East and West Rivers was used to extract surface water data [17].

### Collection and analysis of samples

Sampling was conducted in July and August 2019 in relatively undisturbed areas along the East and West Rivers—areas without agricultural activity, vegetation restoration, or soil disturbance (Table S1). A total of 16 plots (50 × 50 m) were established, with eight plots in each river region. Within each plot, five quadrats (10 × 10 m) were arranged in an X-shaped pattern (Fig. S1), from which soil samples were collected from the 0–20 cm depth. The surface litter layer was removed, and the soil collector was sterilized between samples using deionized water and soil from the same site. Except for undisturbed soil cores used for bulk density (BD) measurements (100 cm³ rings), five subsamples from each quadrat were composited into one representative sample per plot, resulting in 16 composite soil samples. Each sample was divided into two portions: one for physicochemical analysis and the other stored at − 80 °C for DNA extraction and microbial community analysis using next-generation sequencing.

Undisturbed soil cores were oven-dried at 105 °C for 24 h to determine BD. Soil particle size distribution (sand, silt, clay) was measured using a laser diffraction particle size analyzer (Malvern Mastersizer 2000, UK) on air-dried, < 2 mm sieved samples. Soil texture classification followed the USDA system [19]. Physicochemical properties were measured using standard procedures described in [20], including potassium dichromate oxidation for soil organic matter (SOM), alkali diffusion for available nitrogen (AN), the molybdenum blue method for available phosphorus (AP), and atomic absorption spectroscopy for available potassium (AK).

### Sequencing for microbial communities and bioinformatics

Total genomic DNA was extracted using the CTAB/SDS method. The 16 S rRNA and ITS regions were amplified using specific primers (16 S V4: 515 F–806R; ITS1: ITS5–1737 F) with barcodes. PCR products were mixed with 1× loading buffer (containing SYBR Green) and verified via electrophoresis on a 2% agarose gel. Sequencing was performed by Novogene (Beijing, China) using the TruSeq^®^ DNA PCR-Free Library Preparation Kit (Illumina, USA), following the manufacturer’s protocols. Libraries were quality-checked using a Qubit^®^ 2.0 Fluorometer (Thermo Scientific) and the Agilent Bioanalyzer 2100, and sequenced on an Illumina NovaSeq platform to generate 250 bp paired-end reads.

Raw reads were quality filtered using QIIME (v1.9.1) to obtain high-quality clean tags [21]. Sequences were clustered into operational taxonomic units (OTUs) at ≥ 97% similarity using Uparse (v7.0.1001) [22], and representative sequences were selected for taxonomic annotation. Taxonomic classification was performed using the Mothur method and the SILVA132 database [23, 24]. Columnar stacked bar plots were used to display the microbial community composition at the phylum level; unclassified phyla and those with relative abundances < 0.1% were grouped as “Others.” Functional prediction for bacterial and archaeal OTUs was conducted using the FAPROTAX database [25], while fungal OTUs were annotated using FUNGuild [26]. Functional profiles were inferred through the Novogene Cloud Platform.

### Statistical analyses

The Theil–Sen trend analysis was applied to assess long-term trends in annual streamflow and surface water area from 2000 to 2019, combined with the Mann–Kendall non-parametric test, following the method of Basistha et al. [27].

Microbial alpha diversity (richness, evenness, Shannon index) was compared between river regions using the Kruskal–Wallis test. Principal coordinate analysis (PCoA) based on Bray–Curtis dissimilarity was used to visualize microbial community differences, with Permutational multivariate analysis of variance (PERMANOVA) applied to test their significance. Redundancy analysis (RDA) was used to examine relationships between microbial community composition and environmental variables [28]. The RDA was a constrained ordination method that uses linear regression to correlate a distance matrix with explanatory variables. Hydrological indices—mean annual runoff, surface water area (Wa), and seasonal variation in runoff (SeasonCV)—were used as explanatory variables based on 20-year averages. The envfit function tested the significance of these variables. Given that microbial data may deviate from normality, Mantel tests with 999 permutations using Pearson correlations were performed to validate RDA results. Above analyses were performed using the ‘vegan’ package in R [29].

Microbial co-occurrence networks were constructed using Spearman correlations (|r| ≥ 0.6, *p* < 0.05), computed with the corr.test function in the psych R package [30]. Networks were visualized using the igraph R package and Gephi (v0.10.0) [31]. Network metrics—including nodes, edges, degree, clustering coefficient, modularity, and graph density—were calculated in Gephi. Network robustness was assessed by evaluating changes in natural connectivity after node removal. The larger shifts upon the same proportion indicated that there are less robustness or stability within microbial networks. Each node plays a distinct topological role within the network. Topological roles of OTUs were classified as peripherals, connectors, module hubs, or network hubs based on intra-module (Zi) and inter-module (Pi) connectivity [32].

## Results

### Hydrological characteristics

**Fig. 2:**
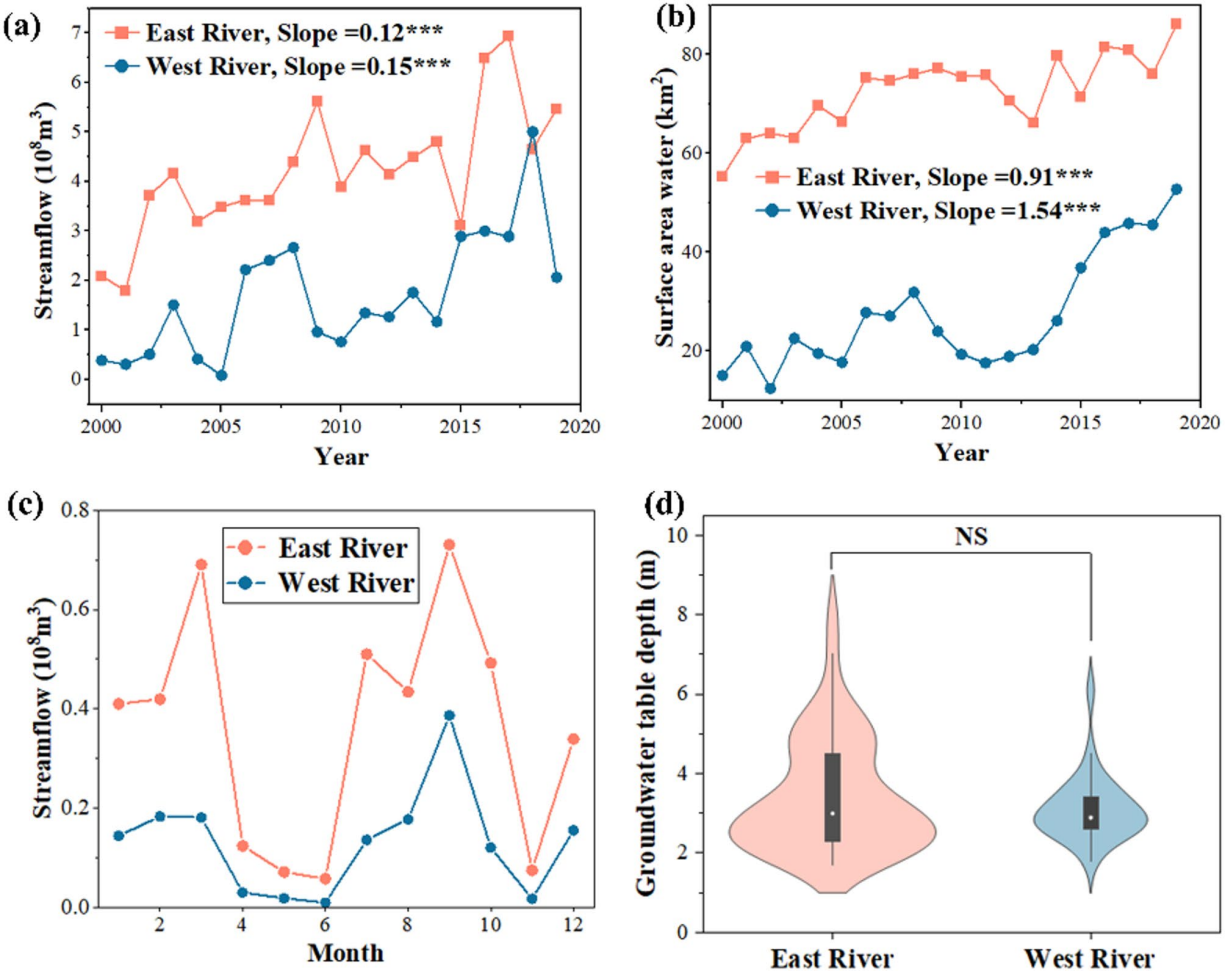
Temporal variations of (**a**) annual streamflow and (**b**) surface water area from 2000 to 2019; (**c**) intra-annual distribution of monthly streamflow and (**d**) the groundwater depth between the East River and the West River in 2019. *, **, and *** indicate that difference is significant at 0.05, 0.01, and 0.001 levels, test by Mann-Kendall test. NS in (D) indicate the no statistical significance were tested by one-way analysis of variance

From 2000 to 2019, both the East and West Rivers exhibited an increasing trend in streamflow (Fig. 2a). Although streamflow in the East River consistently surpassed that in the West River (Table S2), the Theil–Sen slope for the West River (0.15, *P* < 0.001) was steeper than that for the East River (0.12, *P* < 0.001). This increase in streamflow was accompanied by an expansion in surface water area (Fig. 2b). While the East River maintained a significantly larger surface water area than the West River, the rate of increase was higher in the West River, with slopes of 1.54 km²/year (*P* < 0.001) and 0.91 km²/year (*P* < 0.001), respectively. Notably, surface water area in the West River expanded from 15.03 km² in 2000 to 52.69 km² in 2019.

The intra-annual distribution of streamflow showed a low-flow period from April to June, with peaks in March and between July and September (Fig. 2c). The lowest flows occurred in June and November, whereas the highest were recorded in September. The East River exhibited greater monthly streamflow variability than the West River, with coefficients of variation of 0.67 and 0.37, respectively (Table S2). Groundwater depth in 2019 showed no significant difference between the two rivers, averaging approximately 3 m in both regions (Fig. 2d).

### Soil physical-chemical properties

**Fig. 3:**
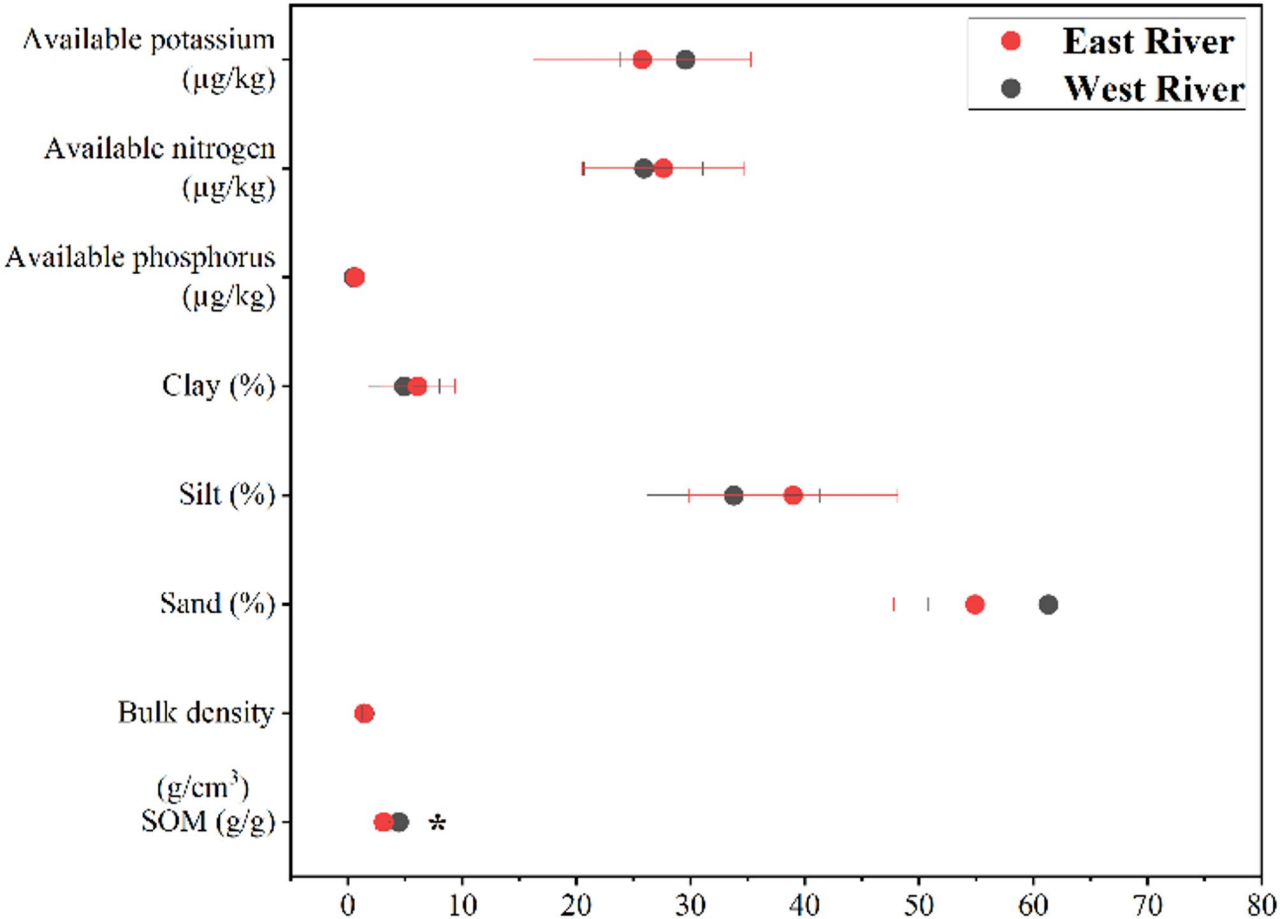
Soil physical-chemical properties between the East River and West River. * indicates that difference is significant at 0.05 level, tested by one-way analysis of variance. The East River and the West River indicated the high-flow and low-flow conditions, respectively

Soil physicochemical properties significantly differed between the East and West River regions (Fig. 3, Table S3). The East River exhibited significantly higher soil organic matter content. BD did not differ significantly between the two regions. Particle size analysis revealed more sand in the East River, while the West River had greater proportions of silt and clay. Among soil nutrients, AP was higher in the West River, whereas AN was slightly, but not significantly, greater in the West River. In contrast, AP was marginally higher in the East River.

### Soil microbial community characteristics

#### Microbial community composition and diversity

**Fig. 4:**
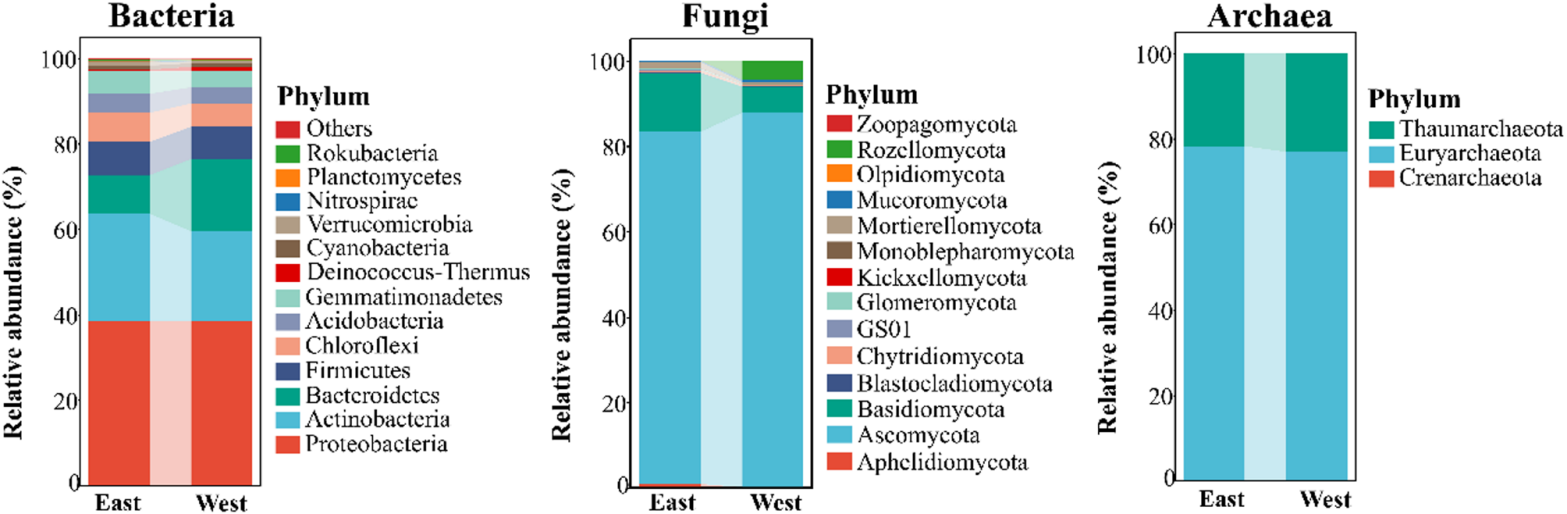
Phyla distribution of the OTUs between the East River and the West River. Phyla abundances less than 0.1% in all samples were combined as ‘Others’. The East River and the West River indicated the high-flow and low-flow conditions, respectively

In total, 9,722 bacterial, 2,303 fungal, and 232 archaeal OTUs were identified. Over 74% of bacterial OTUs belonged to Proteobacteria, Actinobacteria, and Bacteroidetes (Fig. 4a), while more than 85% of fungal OTUs were assigned to Ascomycota (Fig. 4b). Archaeal OTUs were exclusively classified under Thaumarchaeota and Euryarchaeota (Fig. 4c). Microbial richness increased with streamflow (Fig. S2). However, no significant differences were observed in richness, diversity, or evenness between the East and West Rivers, nor were there significant differences in dominant taxon abundance. PCoA revealed clear separation of bacterial communities between the two rivers (*P* < 0.05), but fungal and archaeal communities showed no significant separation (Fig. S3).

#### Co-occurrence networks

**Fig. 5:**
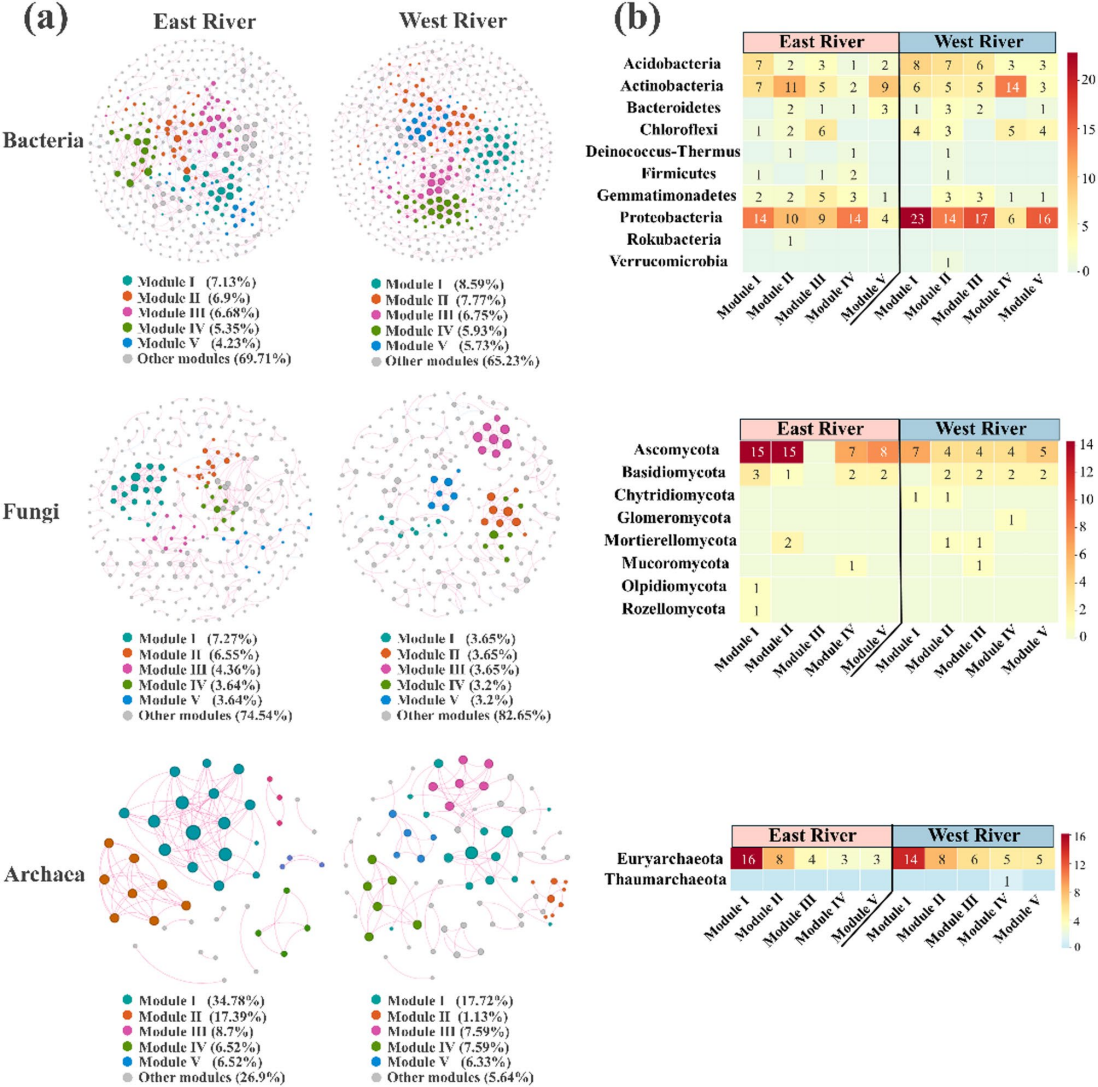
Co-occurrence networks of soil microbial communities at the OTU level between the East River and the West River. **a** In microbial networks, the nodes correspond to the present OTUs colored according to module level. The size of each node corresponds to its degree of connection (the number of edges associated with the node). The edges represent significant Spearman correlations (*R* > 0.60 and *P* < 0.05), which red lines indicate as positive correlations, and blue lines indicate as negative correlations. **b** The heatmap showed the co-occurrence network module corresponds to nodes at phylum level. The East River and the West River indicated the high-flow and low-flow conditions, respectively

All microbial networks had modularity indices exceeding 0.5 (Table S4), with a higher frequency of positive than negative correlations (Fig. 5). In the East River, fungi and archaea exhibited higher average degree values, while bacteria had lower values (Table S4). In contrast, the West River had greater modularity in fungal and archaeal networks. Bacterial networks were primarily composed of nodes from Proteobacteria and Actinobacteria (Table S5). Ascomycota dominated fungal networks. Archaeal networks in the East River were exclusively Euryarchaeota, while 96.2% of archaeal nodes in the West River were from the same phylum. A module hub (OTU 1251, Ascomycota: *Hyaloseta*) and a connector (OTU 4073, Actinobacteria: *Nocardioides*) were detected in the East River network (Fig. S4, Table S6). In the West River, two module hubs (OTU 336 and OTU 796) from Proteobacteria and two connectors (OTU 1637 and OTU 3135) from Actinobacteria and Proteobacteria were identified.

**Fig. 6:**
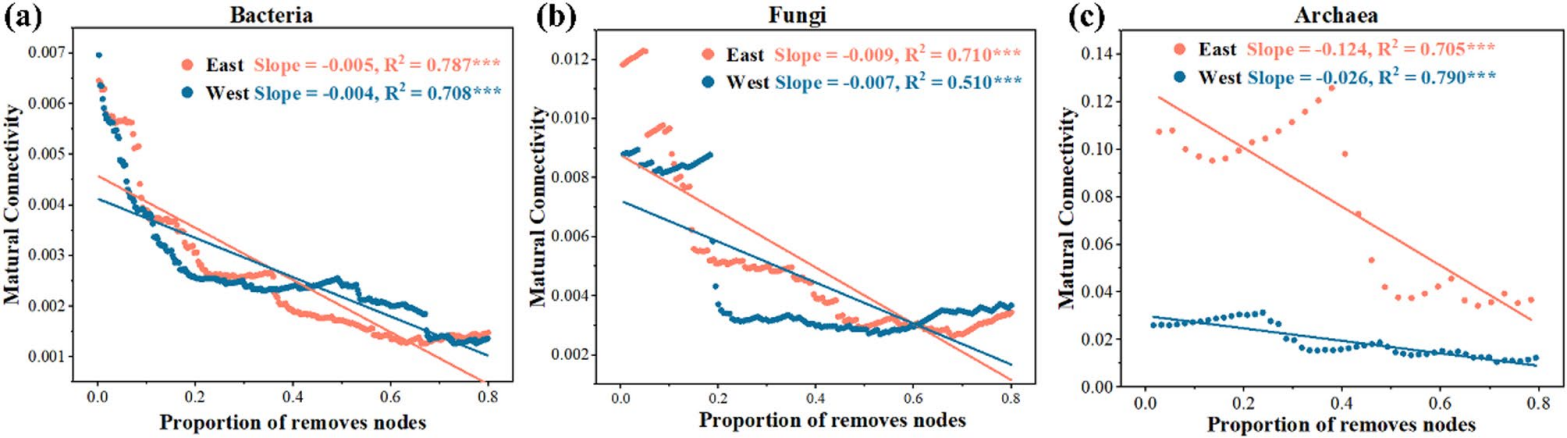
Robustness analysis of networks for bacteria (a), fungi (b), and archaea (c) between the East River and the West River. The East River and the West River indicated the high-flow and low-flow conditions, respectively. Robustness analysis is shown as the relationships between microbial natural connectivity and the proportion of removed nodes, such that larger shifts upon the same proportion indicate that there are less robustness or stability within microbial networks. *, **, and *** indicate that difference is significant at 0.05, 0.01, and 0.001 levels using t-test

Mutual connectivity of microbial communities varied by river (Fig. 6). In bacteria (Fig. 6a), natural connectivity declined with increasing node removal, with slopes of − 0.005 (*R²* = 0.78) and − 0.004 (*R²* = 0.78) in the East and West Rivers, respectively (*P* < 0.001). Fungal networks followed a similar trend (Fig. 6b), with a steeper slope in the East River (− 0.009, *R²* = 0.71) compared to the West River (− 0.007, *R²* = 0.51). Archaeal networks (Fig. 6c) showed the most pronounced decline in the East River (slope = − 0.124, *R²* = 0.71), while the West River had a gentler decline (− 0.026, *R²* = 0.79), indicating greater network stability in the latter.

#### Functional profiles

**Fig. 7:**
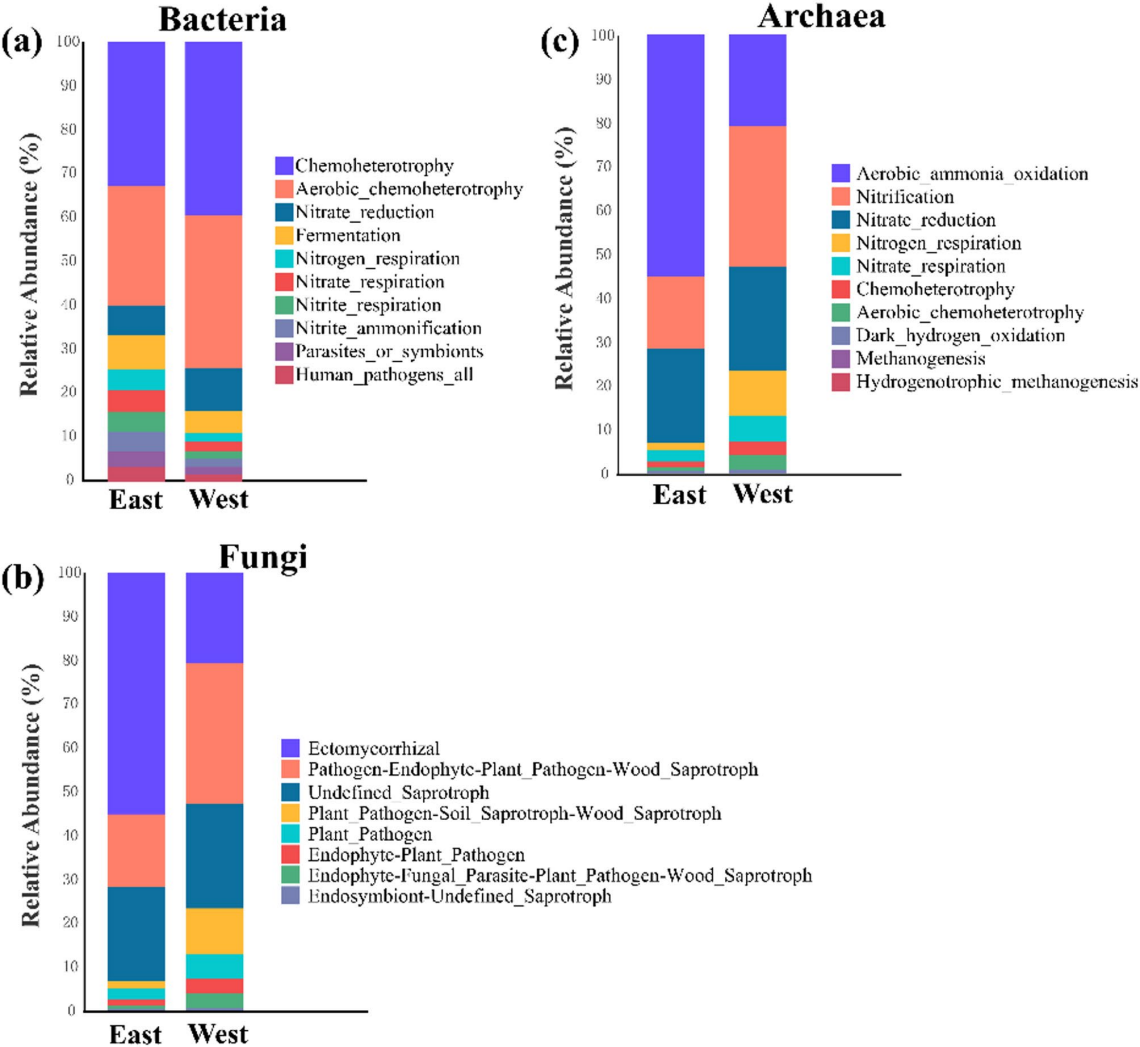
The Barplot representing functional groups of bacteria (a), fungi (b), and archaea (c) with relative abundance in the top 10 functional groups. The microbial functional groups of soil samples between the East River and the West River. The East River and the West River indicated the high-flow and low-flow conditions, respectively

For bacterial community (Fig. 7a), the top three predicted functions were Chemoheterotrophy, Aerobic Chemoheterotrophy, and Nitrate reduction. Functions such as fermentation, nitrogen respiration, and nitrate/nitrite-related pathways were more abundant in the East River. In contrast, the West River showed higher abundances of chemoheterotrophy and nitrate reduction. For fungal community (Fig. 7b), the top three predicted functions were Ectomycorrhizal, Pathogens-Endophyte, and Undefined saprotrophs. Ectomycorrhizal were more prevalent in the East River, while Pathogens-Endophyte, Undefined saprotrophs, and Plant pathogens were more abundant in the West River. For archaeal community (Fig. 7c), the top three predicted functions were Aerobic ammonia oxidation, Nitrification, and Nitrate reduction. Archaeal functions showed greater Aerobic ammonia oxidation in the East River, while Nitrification, Nitrogen respiration, and Chemoheterotrophy were more pronounced in the West River.

### Linkage between microbial community and environmental factors

**Fig. 8:**
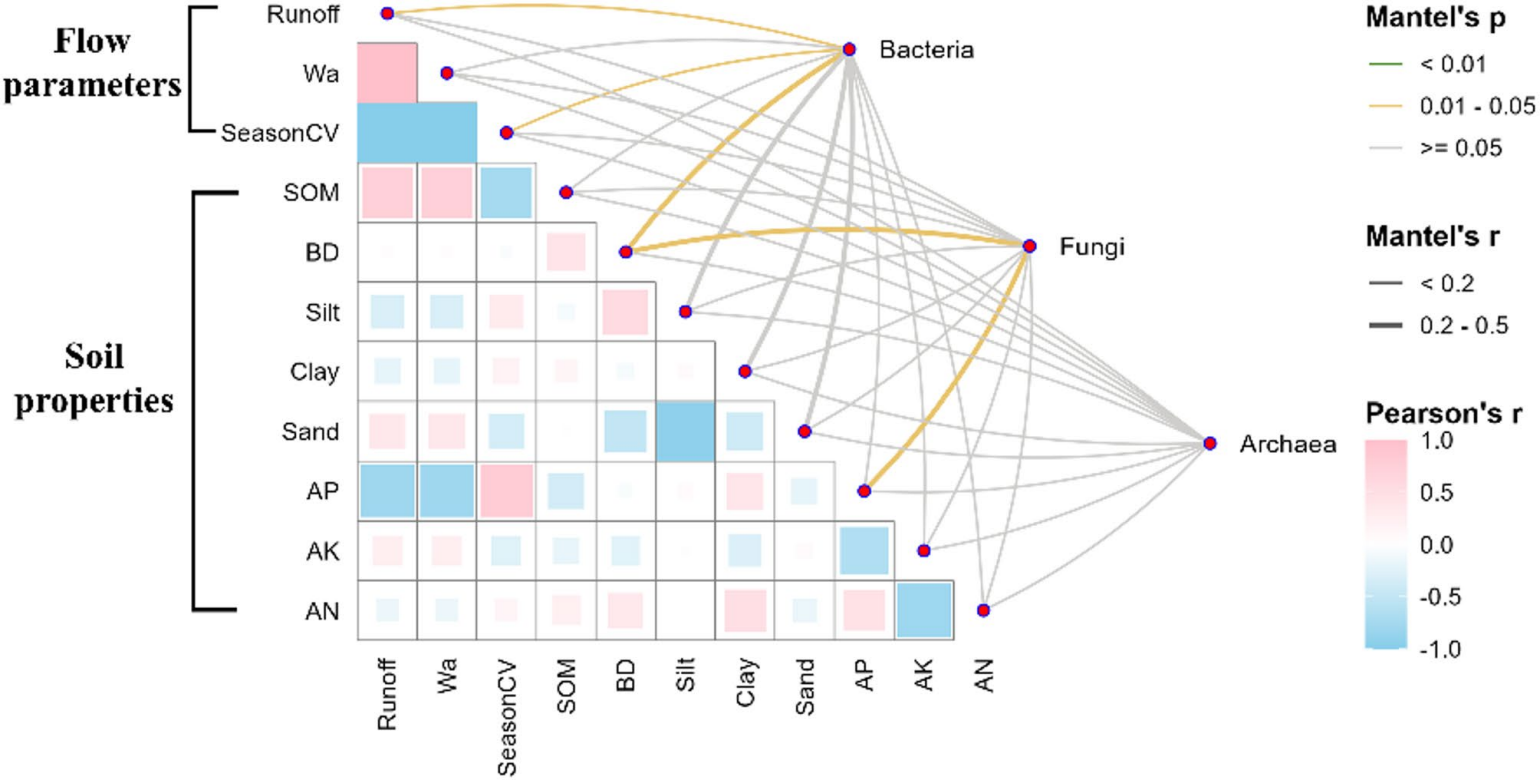
Correlations between environmental factors and microbial community composition. The bacterial, fungal, and archaeal community composition based on Bray–Curtis distance is related to each environmental factor by partial Mantel test. Line width corresponds to the partial Mantel’s r statistic, and line color denotes the statistical significance based on 999 permutations. a color gradient denoting Pearson’s correlation coefficient was also shown, and these factors are synthesized into two groups based on attribute of data surveyed. A color gradient denoted Pearson’s correlation coefficient. Surface water area (Wa), Seasonal variance of runoff (SeasonCV), Soil organic matter (SOM), Bulk density (BD), Available phosphorus (AP), Available nitrogen (AN), and Available potassium (AK)

The RDA model indicated that environmental variables explained 61%, 60%, and 51% of the variation in bacterial, fungal, and archaeal communities, respectively (Fig. S5). Hydrological variables (runoff, Wa, SeasonCV) were moderately correlated (*r* = 0.18–0.48) with microbial community structure. Soil factors such as bulk density and sand content showed stronger correlations (*r* = 0.25–0.66), whereas available potassium had weaker associations (*r* = 0.02–0.15). Partial Mantel tests identified Certain flow parameters (i.e., Runoff, and SeasonCV), and the soil physicochemical factor (i.e., BD) were responsible for bacterial community similarities over environmental distance. (Fig. 8). BD and AP were additionally important for fungi. The dominant environmental drivers varied by flow regime and microbial group (*P* < 0.05): in the East River, BD and AN were key (Fig. 8a), while in the West River, clay content and AK were the main drivers for bacterial and fungal communities, respectively (Fig. 8b).

## Discussion

### Hydro-ecological responses to water diversion in the Heihe river basin

The observed increase in streamflow and surface water area in both the East and West Rivers (Fig. 2) is primarily attributed to the Ecological Water Diversion Project (EWDP), with the East River exhibiting more pronounced high-flow characteristics than the West River [14, 33]. During critical periods, water is preferentially allocated to East Juyan Lake, resulting in greater water delivery to the East River. Under typical conditions, spring ice melt entering the lower Heihe River Basin is initially divided between the two rivers by the Langxinshan Diversion Structure. Water is transmitted via both natural and artificial channels, replenishing groundwater and supporting riparian vegetation throughout the growing season [15, 34]. Notably, from 2000 to 2019, vegetation cover and primary productivity significantly increased in both rivers (Fig. S6).

Soil development in the lower Heihe River Basin is influenced by multiple factors, including Heihe River water, arid climatic conditions, high soil salinity and alkalinity, and the characteristics of local vegetation [35]. The vegetation–hydrology interaction plays a crucial role in soil formation [36], and increased runoff has been shown to enhance total plant cover, thereby promoting organic matter input into soils [37]. This likely explains the direct effect of increased runoff in the East River on vegetation productivity and the indirect positive effect on SOM. However, the parent materials in the lower basin—primarily river alluvium and floodplain deposits—contain high sand content [35]. With the implementation of the water diversion project, increased flow volumes may have further contributed to a higher proportion of sand in the soil (Fig. S7).

Overall, ecosystem conditions in the downstream region of the Heihe River have improved over the past two decades [17]. However, while administrative policies have ensured water delivery, they have failed to sufficiently regulate stakeholder behavior or limit non-ecological water use in the lower reaches. These shortcomings could heighten social tensions between midstream and downstream users, threatening the sustainable development of the basin. Furthermore, the project has contributed to regional imbalances in groundwater availability, leading to vegetation degradation in parts of the middle reaches [14]. Additional degradation in the lower reaches has also occurred due to agricultural expansion and the construction of artificial canals [15]. Hydrological management in the region has thus had substantial effects on local hydrology, ecosystems, and socio-economic structures, with the potential to trigger cascading consequences in water-scarce environments [5, 38].

### Effects of hydrological background and environmental factors on the soil microbial community

The richness of soil microbial diversity is a key determinant of the stability of microbial community structure and function, and it plays a crucial role in evaluating overall soil functionality [39]. The response for the composition of the microbial community in the lower reaches of the Heihe River indicated that the microbial species richness and diversity was not significantly increased with increasing runoff, which was inconsistent with the hypothesis. In previous studies, there were also differences in the responses of microbial community diversity to hydrological regimes. Gionchetta et al. [40] observed shifts in microbial taxonomic groups under varying hydrological conditions that substantially altered surface microbial composition and diversity. In our study, alpha diversity was not significantly influenced by flow conditions (Fig. S2), which may be attributed to microbial adaptations to arid background that buffer their response to changes in flow regimes [41, 42]. A study conducted in the Ain departement (Eastern France) also revealed that microbial diversity did not respond significantly to hydrological regime [37].

Analysis of microbial phyla revealed that dominant groups were consistently prevalent across all soil samples (Fig. 4), echoing findings from dryland ecosystems [42]. Gionchetta et al. [40] also reported increases in taxa such as Thermoplasmata (archaea) and Actinobacteria (bacteria), indicating a shift toward drought-adapted microbial communities. Proteobacteria remained dominant under both high- and low-flow conditions, which was also confirmed by Gionchetta et al. [43], who found Proteobacteria to dominate across all treatments. Prolonged drought has been shown to restructure microbial communities along intermittent riverbanks, favoring taxa adapted to extreme environmental conditions [9].

The dominance of positive associations within microbial co-occurrence networks (Fig. 5) suggests widespread niche similarity and mutualism [44]. These positive interactions may foster syntrophic relationships, enhance nutrient acquisition, or support biofilm formation to mitigate drought stress [44, 45]. Moreover, natural connectivity-based robustness tests indicated that low-flow conditions enhanced microbial network stability (Fig. 6). Microbial responses to environmental stress may involve not only shifts in community composition but also resource reallocation toward acclimation and physiological plasticity [11, 46].

Consistent with findings from other FAPROTAX-based studies [47], chemoheterotrophy was the most dominant predicted bacterial function (Fig. 7). Several studies suggest that microbial composition can influence ecosystem functioning in some cases [48, 49]. In semi-arid desert, Nelson et al. [50] has reported that chemoheterotrophic Proteobacteria might be active nitrogen fixers. Additionally, Thaumarchaeota—ammonia-oxidizing archaea—are prevalent in nutrient-poor soils and play a key role in nitrification [51, 52]. In the present study, it is worth noting that dominant phyla of bacterial and archaeal communities are partially involved in the nitrogen cycle (nitrate reduction, nitrogen respiration, nitrate respiration) and may play a role (nutrient cycling and energy flow) in soil. A significant correlation between AP and fungal communities was also observed, in line with Wang et al. [53], who believed that AP and AN accounted for substantial variance in microbial structure. Variation in microbial responses to hydrological conditions—depending on their physiological breadth—may predictably influence ecosystem function, although specific microbial processes likely respond to differing degrees [48, 49]. Overall, our findings support the hypothesis proposed by Gionchetta et al. [9] that microbial functions respond more strongly to hydrological changes than microbial diversity.

### Limitations

In hydrological studies, spatial resolution is often coarser than temporal resolution, making it difficult to precisely match hydrological data with localized soil sampling points [54], As a result, the study relied on proxy indicators—such as long-term historical runoff—to approximate hydrological influence at the plot scale. However, these proxies may not fully reflect the spatial and temporal heterogeneity of hydrological processes, potentially limiting the accuracy of assessments linking flow regimes to microbial community responses. Temporal variability presents another critical limitation. Soil microbial communities are characterized by rapid turnover and high responsiveness to environmental fluctuations, with temporal variation often exceeding spatial variation [55]. The study was based on a single sampling period during the relatively moist summer season in an arid environment, which may not fully represent year-round microbial dynamics. Lastly, functional profiles of microbial communities were inferred from widely used gene annotation databases (e.g., FAPROTAX, FUNGuild). Functional validation through soil enzyme assays or metabolomic profiling is essential for future studies to determine whether predicted functions align with real ecosystem processes, particularly given the potential presence of the soil N-cycling capacity of these soils [56].

## Conclusion

The present study evaluated the impact of long-term flow change over two decades on multiple soil variables and the microbial community characteristics in the lower Heihe River. Over two decades of water diversion have led to distinct differences in flow magnitude between the East and West Rivers. The combined effect of multiple environmental factors lightly influenced the soil microbial community composition and diversity. Stronger connectivity stability was observed in the West River, particularly within archaeal and fungal networks, accompanied by a higher number of module hubs and connectors. In addition, the bacterial and fungal communities in soil showed a significant relationship with certain flow parameter (runoff and Seasonal variance of runoff), bulk density, and available phosphorus. The majority of the functional prediction of bacterial community was chemoheterotrophy. The bacterial and archaeal communities are involved in the nitrogen cycle such as nitrate reduction, nitrogen respiration and nitrate respiration. Hydrological conditions and available nutrient levels jointly lead to the change of microbial structure and function in arid riparian zones.

## Supplementary Information

Supplementary Material 1: Fig. S1 Soil sampling design in each sample plots. Fig. S2 Differences of microbial richness, Shannon and evenness between the East River and the West River. The East River and the West River indicated the high-flow and low-flow conditions, respectively. Fig. S3 Principal co-ordinate analysis (PCoA) of the microbial communities between the East River and the West River using PERMANOVA test (R2, P). The East River and the West River indicated the high-flow and low-flow conditions, respectively. Fig. S4 Topological roles of soil microbial communities between the East River and the West River. The nodes with either a high value of Zi or Pi were identified as potential keystone taxa, including module hubs (Zi ≥ 2.5, Pi < 0.62), connectors (Zi < 2.5, Pi ≥ 0.62), and network hubs (Zi ≥ 2.5, Pi ≥ 0.62). The East River and the West River indicated the high-flow and low-flow conditions, respectively. Fig. S5 Redundancy analysis (RDA) profiles showing the correlation of environmental factors on bacterial community (a), fungal community (c), and archaeal community (e). The correlation between each environmental factor and the differences in the bacterial community (b), the fungal community (d), and archaeal community (f). The relationship between two environmental factors is positive when their included angle is acute and negative correlation when they are at an obtuse angle. *, **, and *** indicate that difference is significant at 0.05, 0.01, and 0.001 levels using PERMANOVA test. Total explained is environmental factors explained variation in microbial community compositions. Surface water area (Wa), Seasonal variance of runoff (SeasonCV), Soil organic matter (SOM), Bulk density (BD), Available phosphorus (AP), Available nitrogen (AN), and Available kalium (AK). The arrows in RDA indicate the association between environmental factors and microbial community distribution. The longer the arrows, the greater the association and vice versa. The angle between the arrow and ordination axes responds to the association between an environmental factor and the ordination axes. The less the angle, the greater the association and vice versa. Fig. S6 Temporal variations of (a) monthly normalized difference vegetation index (NDVI) and (b) net primary productivity from 2000 to 2019. Asterisks in indicate the statistical significance were tested by Mann-Kendall test (*, **, and *** indicate that difference is significant at 0.05, 0.01, and 0.001 levels). The East River and the West River indicated the high-flow and low-flow conditions, respectively. Fig. S7 Soil texture (Sand, Silt, and Clay) in the lower reaches of the Heihe River with the year (2011 and 2019) and location (East River and West River). Table S1. Sampling sites information. Table S2 Long-term averages of flow Parameters for the East and West Rivers (2000–2019). Table S3 Soil properties between the East River and West River. The bold was significant (*P* <0.05) using one-way analysis of variance. Table S4 Topological characteristics of co-occurrence network of soil microbial communities between the East River and West River. Table S5 The nodes in the co-occurrence network of soil microbial communities at the phylum level between the East River and West River. Table S6 Key nodes of soil microbial network between the East River and the West River. The nodes with either a high value of Zi or Pi were identified as potential keystone taxa, including module hubs (Zi ≥ 2.5, Pi < 0.62), connectors (Zi < 2.5, Pi ≥ 0.62), and network hubs (Zi ≥ 2.5, Pi ≥ 0.62). Table S7 Correlations between environmental factors and microbial community composition for the East River and the West River. The bacterial, fungal, and archaeal community composition based on Bray–Curtis distance is related to each environmental factor by partial Mantel test (r, P). The bold numbers are significant values. *, **, and *** indicate that difference is significant at 0.05, 0.01, and 0.001 levels. Table S8 Soil texture in the lower reaches of the Heihe River with the year (2011 and 2019) and location (the East River and the West River)

## Data Availability

The datasets generated during the current study are available in The National Center for Biotechnology Information (NCBI, https://www.ncbi.nlm.nih.gov/) with BioProject number PRJNA1194339. All data supporting this study are available from the corresponding author upon request.
