# Supplementary material for: Effects of water flow conditions on soil properties and microbial communities in the lower Heihe river Basin, Northwest China
Source: BMC Microbiol. 2025 Oct 7;25:638. doi: 10.1186/s12866-025-04393-7 (PMC12502506; doi:10.1186/s12866-025-04393-7)
Supplement: Supplementary file 1 — Supplementary Material 1: Fig. S1 Soil sampling design in each sample plots. Fig. S2 Differences of microbial richness, Shannon and evenness between the East River and the West River. The East River and the West River indicated the high-flow and low-flow conditions, respectively. Fig. S3 Principal co-ordinate analysis (PCoA) of the microbial communities between the East River and the West River using PERMANOVA test (R2, P). The East River and the West River indicated the high-flow and low-flow conditions, respectively. Fig. S4 Topological roles of soil microbial communities between the East River and the West River. The nodes with either a high value of Zi or Pi were identified as potential keystone taxa, including module hubs (Zi ≥ 2.5, Pi < 0.62), connectors (Zi < 2.5, Pi ≥ 0.62), and network hubs (Zi ≥ 2.5, Pi ≥ 0.62). The East River and the West River indicated the high-flow and low-flow conditions, respectively. Fig. S5 Redundancy analysis (RDA) profiles showing the correlation of environmental factors on bacterial community (a), fungal community (c), and archaeal community (e). The correlation between each environmental factor and the differences in the bacterial community (b), the fungal community (d), and archaeal community (f). The relationship between two environmental factors is positive when their included angle is acute and negative correlation when they are at an obtuse angle. *, **, and *** indicate that difference is significant at 0.05, 0.01, and 0.001 levels using PERMANOVA test. Total explained is environmental factors explained variation in microbial community compositions. Surface water area (Wa), Seasonal variance of runoff (SeasonCV), Soil organic matter (SOM), Bulk density (BD), Available phosphorus (AP), Available nitrogen (AN), and Available kalium (AK). The arrows in RDA indicate the association between environmental factors and microbial community distribution. The longer the arrows, the greater the association and vice versa. The angle b [file 12866_2025_4393_MOESM1_ESM.docx]

**Supplementary Information for**

Effects of water flow conditions on soil properties and microbial communities in the lower Heihe River Basin, Northwest China

**Figures and Tables**


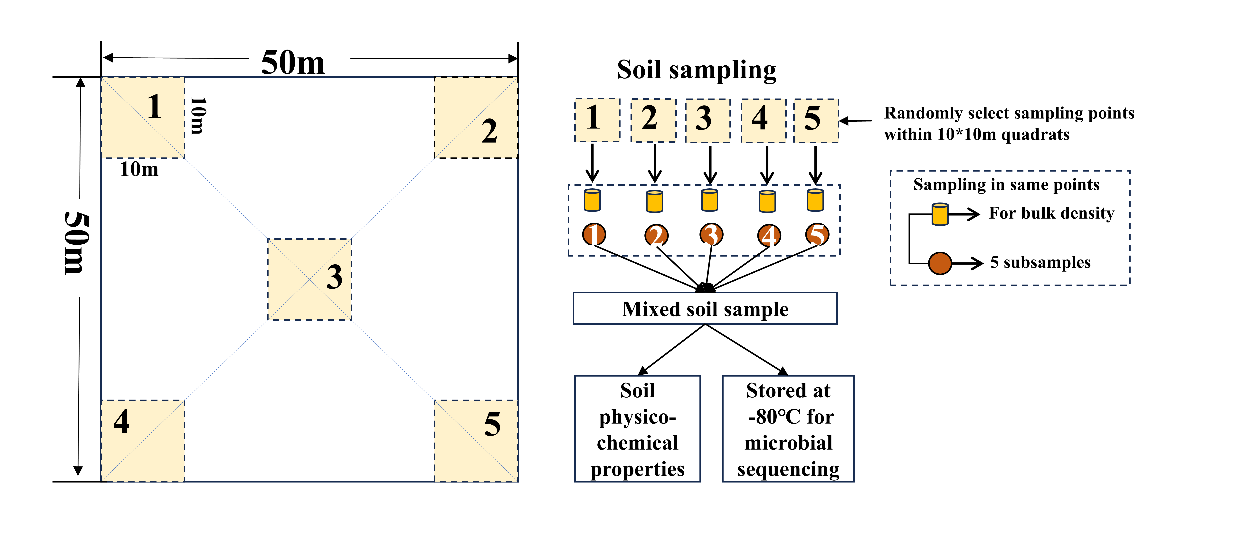


**Fig. S1 Soil sampling design in each sample plots.**


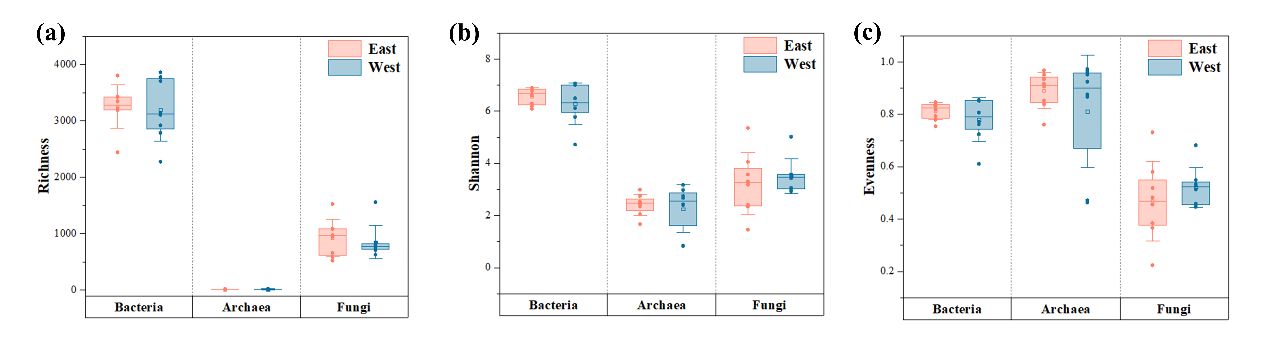


**Fig. S2 Differences of microbial richness, Shannon and evenness between the East River and the West River.** The East River and the West River indicated the high-flow and low-flow conditions, respectively.


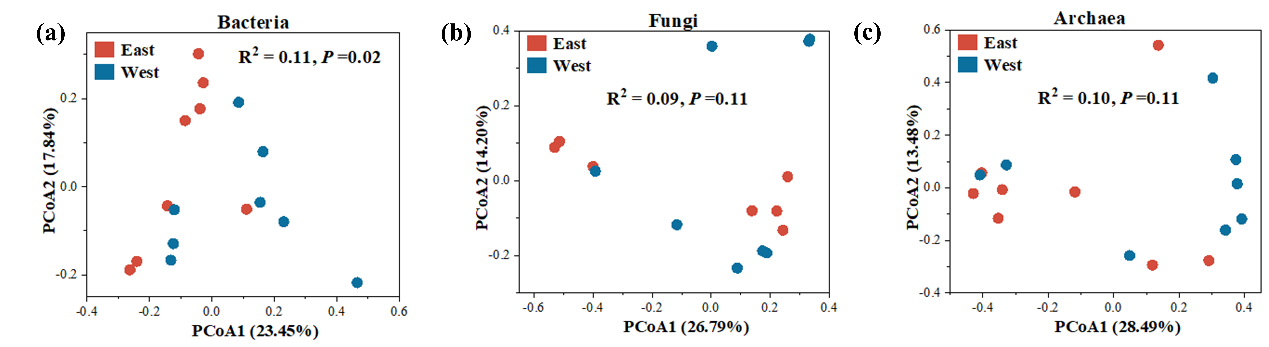


**Fig. S3** **Principal co-ordinate analysis (PCoA) of the microbial communities between the East River and the West River using PERMANOVA test (R^2^, *P*).** The East River and the West River indicated the high-flow and low-flow conditions, respectively.


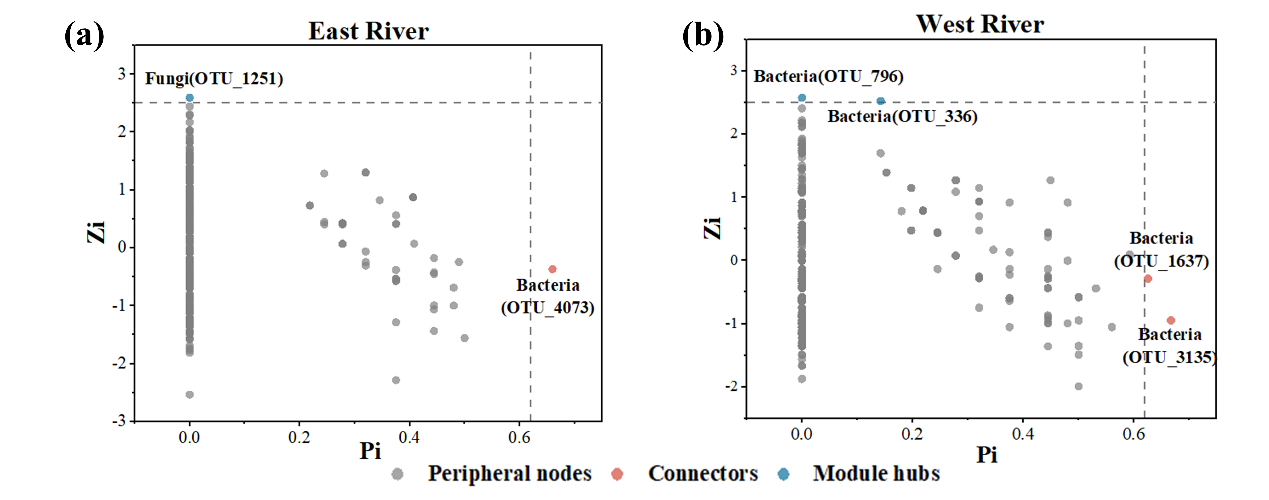


**Fig. S4** **Topological roles of** **soil microbial communities between the East River and the West River. T**he nodes with either a high value of Zi or Pi were identified as potential keystone taxa, including module hubs (Zi ≥ 2.5, Pi < 0.62), connectors (Zi < 2.5, Pi ≥ 0.62), and network hubs (Zi ≥ 2.5, Pi ≥ 0.62). The East River and the West River indicated the high-flow and low-flow conditions, respectively.


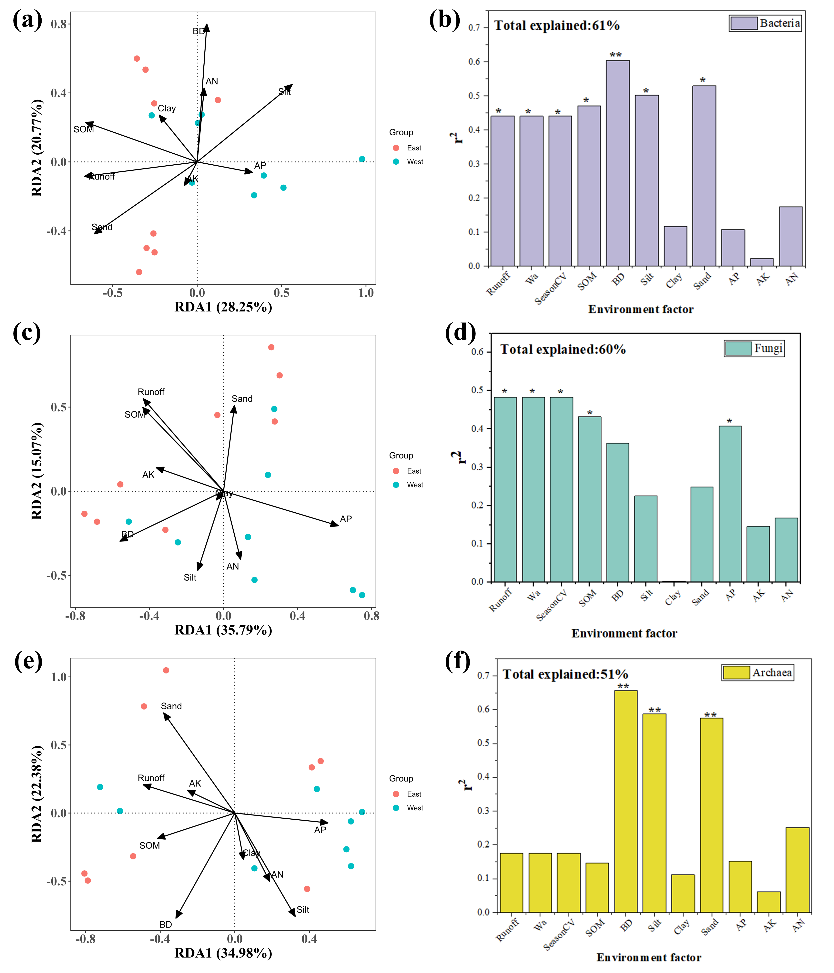


**Fig. S5** **Redundancy analysis (RDA) profiles showin****g the correlation of environmental factors on bacterial community (a), fungal community (c), and archaeal community (e). The correlation between each environmental factor and the differences in the bacterial community (b), the fungal community (d), and archaeal community (f).** The relationship between two environmental factors is positive when their included angle is acute and negative correlation when they are at an obtuse angle. *, **, and *** indicate that difference is significant at 0.05, 0.01, and 0.001 levels using PERMANOVA test. Total explained is environmental factors explained variation in microbial community compositions. Surface water area (Wa), Seasonal variance of runoff (SeasonCV), Soil organic matter (SOM), Bulk density (BD), Available phosphorus (AP), Available nitrogen (AN), and Available kalium (AK). The arrows in RDA indicate the association between environmental factors and microbial community distribution. The longer the arrows, the greater the association and vice versa. The angle between the arrow and ordination axes responds to the association between an environmental factor and the ordination axes. The less the angle, the greater the association and vice versa.


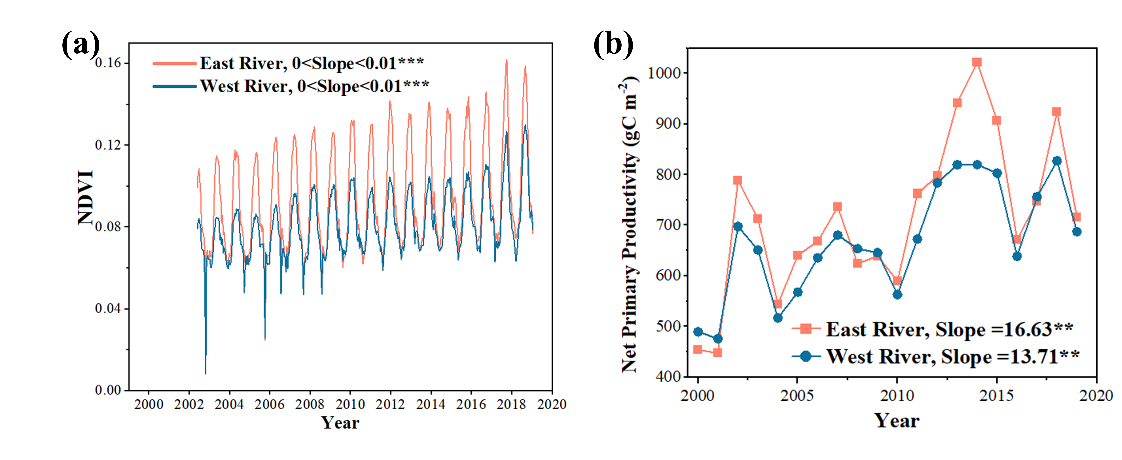


**Fig. S6 Temporal variations of (a) monthly normalized difference vegetation index (NDVI) and (b) net primary productivity from 2000 to 2019.** Asterisks in indicate the statistical significance were tested by Mann-Kendall test (*, **, and *** indicate that difference is significant at 0.05, 0.01, and 0.001 levels). The East River and the West River indicated the high-flow and low-flow conditions, respectively.


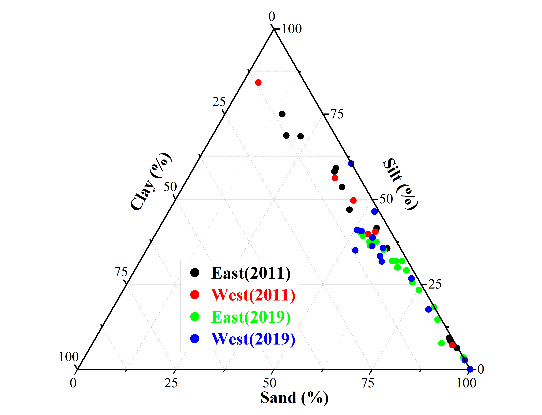


**Fig. S7 Soil texture (Sand, Silt, and Clay) in the lower reaches of the Heihe River with the year (2011 and 2019) and location (East River and West River).**

**Table S1 Sampling sites information.**

| **Location** | **Sampling plot** | **Longitude (°E)** | **Latitude (°N)** | **Sampling time** |
| --- | --- | --- | --- | --- |
| East River  (High-flow) | E1 | 101.20 | 41.99 | July 24th - 29th, 2019 |
|  | E2 | 101.21 | 42.00 |  |
|  | E3 | 101.21 | 41.99 |  |
|  | E4 | 101.22 | 42.00 |  |
|  | E5 | 101.24 | 42.03 |  |
|  | E6 | 101.24 | 42.04 |  |
|  | E7 | 101.25 | 42.03 |  |
|  | E8 | 101.26 | 42.04 |  |
| West River  (Low-flow) | W1 | 100.60 | 41.88 | July 30th - August 9th, 2019 |
|  | W2 | 100.61 | 41.88 |  |
|  | W3 | 100.61 | 41.88 |  |
|  | W4 | 100.61 | 41.88 |  |
|  | W5 | 100.61 | 41.91 |  |
|  | W6 | 100.62 | 41.94 |  |
|  | W7 | 100.62 | 41.94 |  |
|  | W8 | 100.62 | 41.95 |  |

**Table S2 Long-term averages of flow Parameters for the East and West Rivers (2000–2019).**

| Parameter | East River  (High-flow) | West River  (Low-flow) | One-way analysis of variance |
| --- | --- | --- | --- |
| Runoff (10^8^m^3^) | 4.22±1.25 | 1.68**±**1.19 | *P* < 0.001 |
| Surface water area (10^7^m^3^) | 7.24**±**0.74 | 2.73**±**1.14 | *P* < 0.001 |
| SeasonCV | 0.62 | 0.79 |  |

**Table S3 Soil properties between the East River and West River.** The bold was significant (*P* <0.05) using one-way analysis of variance.

| Parameter | East River  (High-flow) | West River  (Low-flow) |
| --- | --- | --- |
| SOM (g/g) | **4.46****±0.63** | **3.13±0.69** |
| Bulk density (g/cm^3^) | 1.43±0.14 | 1.42±0.11 |
| Sand (%) | 61.31±10.52 | 54.91±7.13 |
| Silt (%) | 33.78±7.57 | 38.99±9.14 |
| Clay (%) | 4.91±3.12 | 6.10±3.27 |
| Available phosphorus (μg/kg) | 0.47±0.07 | 0.66±0.15 |
| Available nitrogen (μg/kg) | 25.89±5.19 | 27.63±7.10 |
| Available kalium (μg/kg) | 29.55±5.68 | 25.77±9.53 |

**Table S4 Topological characteristics of co-occurrence network of soil microbial communities between the East River and West River.**

| Network indexes | Bacteria | | Fungi | | Archaea | |  |
| --- | --- | --- | --- | --- | --- | --- | --- |
|  | East River  (High-flow) | West River  (Low-flow) | East River  (High-flow) | West River  (Low-flow) | East River  (High-flow) | West River  (Low-flow) | |
| Total nodes | 449 | 489 | 275 | 219 | 46 | 79 | |
| Total links (positive – negative) | 617(465–152) | 783 (743–40) | 356(339–17) | 223(209–14) | 112(112–0) | 107(107–0) | |
| Average degree | 2.75 | 3.20 | 2.59 | 2.04 | 4.87 | 2.71 | |
| Average clustering coefficient | 0.49 | 0.43 | 0.69 | 0.81 | 0.89 | 0.86 | |
| Average path distance | 6.99 | 6.75 | 2.70 | 1.35 | 1.40 | 1.74 | |
| Modularity | 0.88 | 0.83 | 0.92 | 0.96 | 0.59 | 0.88 | |
| Graph density | 0.01 | 0.01 | 0.01 | 0.01 | 0.11 | 0.04 | |

**Table S5 The nodes in the co-occurrence network of soil microbial communities at the phylum level between the East River and West River.**

| Community | Phylum | Nodes number in each phylum | |
| --- | --- | --- | --- |
|  |  | East River  (High-flow) | West River (Low-flow) |
| Bacteria | Proteobacteria | 173 | 194 |
|  | Actinobacteria | 102 | 115 |
|  | Acidobacteria | 38 | 47 |
|  | Chloroflexi | 33 | 42 |
|  | Bacteroidetes | 31 | 36 |
|  | Gemmatimonadetes | 34 | 27 |
|  | Firmicutes | 32 | 18 |
|  | Deinococcus-Thermus | 4 | 4 |
|  | Nitrospirae | 1 | 2 |
|  | Verrucomicrobia |  | 2 |
|  | Cyanobacteria |  | 1 |
|  | Rokubacteria | 1 | 1 |
| Fungi | Ascomycota | 205 | 164 |
|  | Basidiomycota | 44 | 33 |
|  | Mortierellomycota | 8 | 8 |
|  | Chytridiomycota | 4 | 4 |
|  | Mucoromycota | 4 | 5 |
|  | Aphelidiomycota | 2 |  |
|  | Glomeromycota | 2 | 3 |
|  | Olpidiomycota | 2 |  |
|  | Rozellomycota | 2 | 1 |
|  | Kickxellomycota | 1 |  |
|  | Monoblepharomycota | 1 |  |
|  | Blastocladiomycota |  | 1 |
| Archaea | Euryarchaeota | 46 | 76 |
|  | Thaumarchaeota |  | 3 |

**Table S6 Key nodes of soil microbial network between the East River and the West River.** The nodes with either a high value of Zi or Pi were identified as potential keystone taxa, including module hubs (Zi ≥ 2.5, Pi < 0.62), connectors (Zi < 2.5, Pi ≥ 0.62), and network hubs (Zi ≥ 2.5, Pi ≥ 0.62).

| Flow condition | Typology | OUT_ID | Phylum | Species |
| --- | --- | --- | --- | --- |
| East River (High-flow) | Module hubs | OUT_1251 | Ascomycota | Hyaloseta |
|  | Connectors | OUT_4073 | Actinobacteria | Nocardioides |
| West River (Low-flow) | Module hubs | OUT_336 | Proteobacteria | Pedomicrobium |
|  | Module hubs | OUT_796 | Proteobacteria | Unclassified |
|  | Connectors | OUT_1637 | Actinobacteria | Unclassified |
|  | Connectors | OUT_3135 | Proteobacteria | Unclassified |

**Table S7 Correlations between environmental factors and microbial community composition for the East River and the West River. The bacterial, fungal, and archaeal community composition based on Bray–Curtis distance is related to each environmental factor by partial Mantel test (r, *P*). The bold numbers are significant values. *, **, and *** indicate that difference is significant at 0.05, 0.01, and 0.001 levels.**

| Soil properties | Bacteria | | Fungi | | Archaea | |  |  |
| --- | --- | --- | --- | --- | --- | --- | --- | --- |
|  | East River  (High-flow) | West River  (Low-flow) | East River  (High-flow) | West River  (Low-flow) | East River  (High-flow) | West River  (Low-flow) | | |
| SOM | 0.20 | -0.08 | 0.22 | 0.11 | 0.11 | 0.31 | |  |
| BD | **0.57 *** | 0.28 | **0.63 *** | -0.06 | 0.21 | -0.04 | |  |
| Silt | -0.09 | 0.65 | 0.00 | -0.12 | 0.19 | -0.02 | |  |
| Clay | 0.00 | **0.60 *** | -0.16 | -0.37 | -0.12 | -0.23 | |  |
| Sand | -0.06 | 0.58 | -0.05 | -0.19 | 0.11 | -0.04 | |  |
| Available phosphorus | 0.12 | 0.22 | -0.07 | 0.28 | -0.17 | 0.10 | |  |
| Available kalium | 0.10 | -0.05 | -0.15 | **0.34 *** | 0.03 | 0.17 | |  |
| Available nitrogen | **0.66 **** | 0.07 | **0.65 *** | 0.24 | **0.34 *** | 0.08 | |  |

**Table S8 Soil texture in the lower reaches of the Heihe River the year (2011 and 2019) and location (the East River and the West River).**

| Flow condition | Year | Sand (%) | Silt (%) | Clay (%) |
| --- | --- | --- | --- | --- |
|  | 2011 | 49.65±27.50 | 44.94±24.07 | 5.41±3.72 |
|  | 2019 | 67.80b±16.71 | 28.36±14.68 | 3.83±3.21 |
| East River  (High-flow) | 2011 | 50.45±28.21 | 44.27±24.63 | 5.28±3.93 |
|  | 2019 | 48.05±28.58 | 46.27±25.15 | 5.68±3.59 |
| West River  (Low-flow) | 2011 | 69.12±14.64 | 27.26±12.32 | 3.62±2.81 |
|  | 2019 | 66.13±19.43 | 29.76±17.58 | 4.11±3.74 |

Note: The soil particle size data for 2011 were obtained from Zhao et al. [1]. The data for 2019 were derived from the survey data of the study.

References:

1. Zhao Y, Feng Q, Yang H. Soil salinity distribution and its relationship with soil particle size in the lower reaches of Heihe River, Northwestern China. Environmental Earth Sciences. 2016;75(9):810; doi: 10.1007/s12665-016-5603-8.
